# Carvedilol versus Metoprolol in Patients with Ventricular Tachyarrhythmias

**DOI:** 10.3390/jcdd9080274

**Published:** 2022-08-16

**Authors:** Tobias Schupp, Michael Behnes, Mohammad Abumayyaleh, Kathrin Weidner, Jonas Rusnak, Kambis Mashayekhi, Thomas Bertsch, Ibrahim Akin

**Affiliations:** 1 First Department of Medicine, University Medical Centre Mannheim (UMM), Faculty of Medicine Mannheim, University of Heidelberg, Theodor-Kutzer-Ufer 1-3, 68167 Mannheim, Germany; 2 Department of Internal Medicine and Cardiology, Mediclin Heart Centre Lahr, 77933 Lahr, Germany; 3 Institute of Clinical Chemistry, Laboratory Medicine and Transfusion Medicine, Nuremberg General Hospital, Paracelsus Medical University, 90419 Nuremberg, Germany

**Keywords:** ventricular tachycardia, ventricular fibrillation, mortality, carvedilol, metoprolol, medical treatment, pharmacological drugs

## Abstract

The study investigates the prognostic role of treatment with carvedilol as compared to metoprolol in patients with ventricular tachyarrhythmias. A large retrospective registry was used including consecutive patients on beta-blocker (BB) treatment with episodes of ventricular tachycardia (VT) or fibrillation (VF) from 2002 to 2015. Patients treated with carvedilol were compared to patients with metoprolol. The primary prognostic outcome was all-cause mortality at three years. Secondary endpoints comprised a composite arrhythmic endpoint (i.e., recurrences of ventricular tachyarrhythmias, appropriate implantable cardioverter defibrillator (ICD) therapies) and cardiac rehospitalization. Kaplan–Meier survival curves, multivariable Cox regression analyses, and propensity score matching were applied for statistics. There were 1098 patients included, 80% treated with metoprolol and 20% with carvedilol. Patients with carvedilol were older, more often presenting with VT (78% vs. 62%; *p* = 0.001) and with more advanced stages of heart failure. Treatment with carvedilol was associated with comparable all-cause mortality compared to metoprolol (20% vs. 16%, log rank *p* = 0.234; HR = 1.229; 95% CI 0.874–1.728; *p* = 0.235). However, secondary endpoints (i.e., composite arrhythmic endpoint: 32% vs. 17%; *p* = 0.001 and cardiac rehospitalization: 25% vs. 14%; *p* = 0.001) were more frequently observed in patients with carvedilol, which was still evident after multivariable adjustment. After propensity score matching (*n* = 194 patients with carvedilol and metoprolol), no further differences regarding the distribution of baseline characteristics were observed. Within the propensity-score-matched cohort, higher rates of the composite arrhythmic endpoint were still observed in patients treated with carvedilol, whereas the risk of cardiac rehospitalization was not affected by the type of beta-blocker treatment. In conclusion, carvedilol and metoprolol are associated with comparable all-cause mortality in patients with ventricular tachyarrhythmias, whereas the risk of the composite arrhythmic endpoint was increased in patients with carvedilol therapy.

## 1. Introduction

The class I A indication for treatment with beta-blockers (BBs) for primary prevention of sudden cardiac death (SCD) predominantly relies on studies suggesting improved outcomes in patients with systolic heart failure (HF) or secondary to acute myocardial infarction (AMI) [1]. Besides improving long-term mortality, a significant reduction of SCD rates was demonstrated for patients treated with different types of BB as compared to patients without [2,3,4]. For instance, the MERIT-HF study demonstrated decreased long-term mortality in 3991 HF patients randomized for metoprolol treatment during a median follow-up of one year, resulting in a lower risk of SCD for patients on metoprolol therapy [2]. However, within the large landmark studies in the field of BBs, patients were usually randomized to receive treatment with BBs or placebo. In contrast, randomized trials or large registries investigating the prognostic value of different types of BBs are limited and restricted to pre-selected subgroups, such as patients with systolic HF or AMI. For instance, the OPTAIN multi-center registry investigated the prognostic value of carvedilol compared to metoprolol in over 5500 patients with AMI, suggesting a comparable risk of all-cause mortality at three years. However, improved survival was seen in patients with carvedilol treatment in the presence of HF with the left ventricular ejection fraction (LVEF) ≤ 40% [5]. In line with this, we recently demonstrated that BB therapy improves survival in patients with ventricular tachyarrhythmias as compared to patients discharged without BB treatment, whereas no further stratification by the type of BB was performed [6].

However, different types of BBs may affect long-term prognosis following ventricular tachyarrhythmias. Compared to metoprolol, carvedilol inhibits the alpha 1 and beta 2 adrenergic receptors in addition to beta 1 inhibition, which may further decrease harmful catecholaminergic effects and lower plasma potassium levels, which both may affect cardiac arrhythmogenicity [7].

To the best of our knowledge, the secondary preventive effect of treatment with metoprolol or carvedilol has not yet been investigated within a “real-life” study. Therefore, the present study evaluates the prognosis of patients treated with carvedilol compared to metoprolol regarding the primary endpoint all-cause mortality and the secondary composite arrhythmic endpoint, as well as the risk of cardiac rehospitalization at three years.

## 2. Materials and Methods

### 2.1. Data Collection and Documentation 

The present study included retrospectively all patients surviving index episodes of ventricular tachyarrhythmias (i.e., ventricular tachycardia (VT) and ventricular fibrillation (VF)) on admission from 2002 until 2015 at our institution, as recently published [7]. The study was derived from an analysis of the “Registry of Malignant Arrhythmia and Sudden Cardiac Death—Influence of Diagnostics and Interventions (RACE-IT)”, a single-center registry including consecutive patients presenting with ventricular tachyarrhythmias and aborted cardiac arrest being acutely admitted to the University Medical Center Mannheim (UMM), Germany (clinicaltrials.gov (accessed on 1 August 2022) identifier: NCT02982473) from 2002 until 2015. The study was carried out according to the principles of the Declaration of Helsinki and was approved by the Medical Ethics Committee II of the Medical Faculty Mannheim, University of Heidelberg, Germany (Ethical Approval Number: 2016-612N-MA). In accordance with local guidelines, consent to participate was not necessary because of the retrospective study design.

### 2.2. Inclusion and Exclusion Criteria

Consecutive patients with BB therapy (i.e., metoprolol or carvedilol) were included. The decision to treat patients with BBs was based on the discretion of the cardiologists during routine care according to the European guidelines [1,8,9,10]. Patients without treatment with metoprolol or carvedilol and patients dying during index hospitalization were excluded from the present study. All other medical therapies apart from BBs were allowed. 

### 2.3. Primary and Secondary Endpoints

The follow-up period was set at three years for all outcomes. The primary prognostic endpoint was all-cause mortality. All-cause mortality was documented using our electronic hospital information system and by directly contacting state resident registration offices (“bureau of mortality statistics”) all across Germany. The identification of patients was verified by place names, surname, day of birth, and registered living addresses. Secondary endpoints were a composite endpoint (i.e., recurrences of ventricular tachyarrhythmias, appropriate ICD therapies) and cardiac rehospitalization. Cardiac rehospitalization comprised rehospitalization due to VT, VF, acute myocardial infarction (AMI), acute heart failure, and inappropriate device therapy.

### 2.4. Further Risk Stratification

Further risk stratification was performed according to the underlying cardiac pathology. Patients with non-AMI, ST segment elevation myocardial infarction (STEMI), non-ST segment elevation myocardial infarction (NSTEMI), ischemic (ICMP) and non-ischemic cardiomyopathy (NICMP), as well as patients with idiopathic ventricular tachyarrhythmias were analyzed.

STEMI was defined as a novel rise in the ST segment in at least two contiguous leads, with ST segment elevation ≥ 2.5 mm in men < 40 years, ≥2 mm in men ≥ 40 years, or ≥1.5 mm in women in leads V2–V3 and/or 1 mm in the other leads. Additional ECG criteria were new ST depression or inversion, T wave alterations, Q waves, or new left bundle branch block [11]. NSTEMI was defined as the presence of an acute coronary syndrome with a troponin I increase above the 99th percentile of a healthy reference population, in absence of ST segment elevation, but with persistent or transient ST segment depression, inversion, or alteration of the T wave, or a normal ECG in the presence of a coronary culprit lesion. The culprit lesion was defined as an acute complete thrombotic occlusion for STEMI and as any relevant critical coronary stenosis for NSTEMI, with the potential need for coronary revascularization either by percutaneous coronary intervention (PCI) or coronary artery bypass grafting (CABG). The presence of a coronary culprit lesion was mandatory for both diagnoses of NSTEMI and STEMI. Evidence of regional wall motion abnormalities was also included in AMI diagnosis, as much as was available. Values of the left ventricular ejection fraction (LVEF) were retrieved from standardized transthoracic echocardiographic examinations, usually performed before hospital discharge in survivors, to assess realistic LVEF values beyond the acute phase of acute coronary ischemia during AMI. In minor part, and only if available, earlier LVEF values, assessed upon admission or during intensive care, were retrieved from patients who died while already within the acute phase of AMI [12].

ICMP comprised all patients with an LVEF < 55% and had either prior documented CAD or newly diagnosed CAD, as well as patients with AMI assessed by coronary angiography at an index stay sufficient to cause myocardial dysfunction. Identification of CAD (defined as at least one relevant stenosis of one epicardial coronary artery of more than 50%) was based on the judgment of the investigating interventional cardiologist during routine care. All coronary angiograms and reports were reassessed post hoc by two independent interventional cardiologists to determine whether the CAD was sufficient for the causality of myocardial dysfunction [13]. NICMP comprised all patients with an LVEF < 55%, in the absence of CAD, valvular heart disease, and congenital heart disease, sufficient to cause the observed myocardial abnormality. The following types were allocated to the NICMP group: dilated cardiomyopathy (DCM), hypertrophic obstructive cardiomyopathy, arrhythmogenic right ventricular dysplasia (ARVD), and noncompaction cardiomyopathy (NCCMP) [13,14,15,16].

Patients presenting without AMI, ICMP, and NICMP and who had no evidence of an impaired LVEF or structural heart disease were classified as patients with “idiopathic ventricular tachyarrhythmias”.

### 2.5. Statistical Methods

Quantitative data are presented as the mean ± the standard error of the mean (SEM), the median and interquartile range (IQR), and the ranges depending on the distribution of the data and were compared using Student’s *t*-test for normally distributed data or the Mann-Whitney *U*-test for nonparametric data. Deviations from a Gaussian distribution were tested by the Kolmogorov–Smirnov test. Spearman’s rank correlation for nonparametric data was used to test univariate correlations. Qualitative data are presented as absolute and relative frequencies and were compared using the Chi² test or Fisher’s exact test, as appropriate. 

Firstly, the univariable Kaplan–Meier method was applied to evaluate prognostic differences within the entire cohort. Secondly, multivariable Cox regression models were developed using the “forward selection” option, where only statistically significant variables (*p* < 0.05) were included and analyzed simultaneously. Predefined variables used for multivariable Cox regressions included: baseline parameters (age, male gender), chronic diseases (chronic kidney disease, diabetes mellitus), prior heart failure, AMI, atrial fibrillation (AF), CAD, an LVEF < 35%, and carvedilol versus metoprolol therapy. Multivariable Cox regression analyses were performed within the entire study cohort, as well as within important subgroups.

Finally, propensity score matching was applied. Propensity scores (1:1) were created for the comparisons of carvedilol versus metoprolol, including the entire study cohort and applying a non-parsimonious multivariable logistic regression model. Propensity scores were created according to the presence of the following independent variables: age, sex, diabetes, chronic kidney disease, prior heart failure, CAD, LVEF, CPR, index ventricular tachyarrhythmia (i.e., VT/VF), and presence of an ICD. Based on the propensity score values counted by logistic regression, for each patient, one patient in the control group with a similar propensity score value was found (accepted difference of propensity score value: <5%). Univariable stratification was performed using the Kaplan–Meier method, with comparisons between groups using univariable hazards ratios (HRs) given together with 95% confidence intervals.

The result of a statistical test was considered significant for *p* < 0.05. SPSS (Version 25, IBM, Armonk, New York, NY, USA) was used for statistics. 

## 3. Results

### 3.1. Study Population

**Figure 1:**
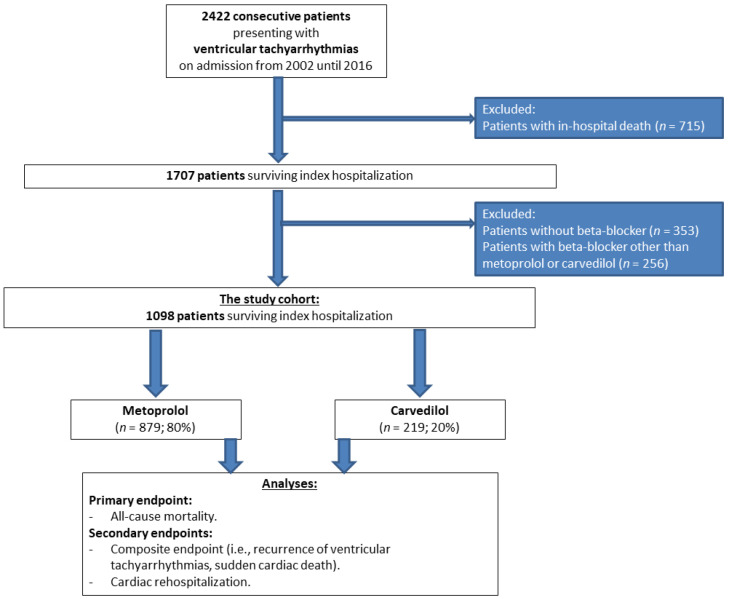
Flow chart of the study population.

From a total of 2422 patients with ventricular tachyarrhythmias, 715 were excluded for in-hospital death, 353 without BB treatment, and 259 patients with BB treatment other than metoprolol or carvedilol (Figure 1; flow chart). The final study cohort comprised 1098 patients with metoprolol (*n* = 879; 80%) with a mean daily dosage of 76.8 mg (±1.1 mg) or carvedilol (*n* = 219; 20%) with a mean daily dosage of 20.9 mg (±1.0 mg). 

As seen in Table 1 (left panel), patients had a median age of 67 years, and most patients were males (73–80%). An index episode of VT vs. VF was more common in patients treated with carvedilol (78% vs. 62%; *p* = 0.001). Cardiovascular risk factors, especially diabetes mellitus (37% vs. 25%; *p* = 0.001) and hyperlipidemia (39% vs. 31%; *p* = 0.020), were more common in the carvedilol group. Especially the rates of prior myocardial infarction, coronary artery disease, and chronic heart failure were higher in patients with carvedilol (*p* ≤ 0.02). In line with this, more patients with carvedilol had an LVEF < 35% (63% vs. 29%; *p* = 0.001). Finally, treatment rates with angiotensin receptor blockers, amiodarone, digitalis, and aldosterone antagonists were more frequently observed in the carvedilol group (*p* ≤ 0.019) (Table 1).

### 3.2. Follow-Up Data and Primary and Secondary Endpoints within the Entire Study Cohort

**Figure 2:**
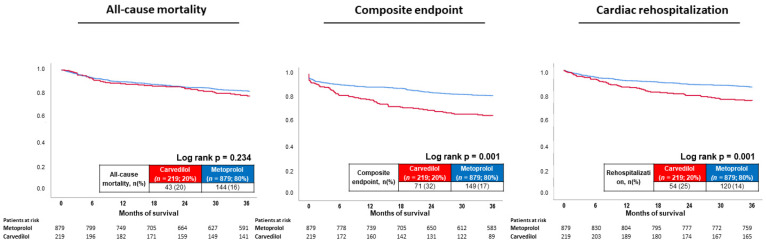
Prognostic impact of metoprolol versus carvedilol treatment on all-cause mortality (**left**), risk of the composite endpoint (i.e., recurrence of ventricular tachyarrhythmias, sudden cardiac death) (**middle**), and cardiac rehospitalization (**right**) within the entire study cohort.

The median follow-up time within the entire study cohort was 4.8 years (IQR 2.3–8.3 years). At three years of follow-up, the primary endpoint all-cause mortality occurred in 20% of the patients with carvedilol treatment and in 16% with metoprolol. Accordingly, the risk of all-cause mortality was not affected by the type of BB (log rank p = 0.234; HR = 1.229; 95% CI 0.874–1.728; *p* = 0.235) (Table 2 and Figure 2, left panel). A comparable effect on all-cause mortality in patients treated with carvedilol and metoprolol was seen at 1 year (HR = 0.861; 95% CI 0.547–1.355; *p* = 0.517) and 2 years of follow-up (HR = 0.918; 95% CI 0.622–1.356; *p* = 0.667).

In contrast, carvedilol was associated with higher rates of the composite endpoint (32% vs. 17%; log rank p = 0.001; HR = 2.128; 95% CI 1.604–2.824; *p* = 0.001), which was already seen after 1 year (HR = 2.067; 95% CI 1.452–2.941; *p* = 0.001) and 2 years of follow-up (HR = 2.132; 95% CI 1.577–2.881; *p* = 0.001).

Finally, the risk of cardiac rehospitalization at three years was increased in patients with carvedilol therapy (25% vs. 14%; log rank p = 0.001; HR = 1.908; 95% CI 1.384–2.631; *p* = 0.001), which was already evident after 1 year of follow-up (HR = 1.641; 95% CI 1.075–2.507; *p* = 0.022) and 2 years of follow-up (HR = 1.846; 95% CI 1.300–2.621; *p* = 0.001) (Figure 2, middle and right panel). In both groups, acute HF was the most common reason for cardiac rehospitalization, whereas the reasons for rehospitalization did not differ among patients with carvedilol and metoprolol (Table 2).

### 3.3. Multivariable Cox Regression Models

After multivariable adjustment, the type of BB therapy (i.e., carvedilol vs. metoprolol) was not associated with all-cause mortality at three years (HR = 0.811; 95% CI 0.550–1.194; *p* = 0.288) (Table 3). In contrast, especially increasing age (HR = 1.522; *p* = 0.001), the presence of diabetes mellitus (HR = 1.992; *p* = 0.001), and chronic kidney disease (HR = 1.721; *p* = 0.001) were associated with impaired long-term mortality. However, carvedilol was associated with increased risk of the composite endpoint (HR = 1.726; 95% CI 1.261–2.364; *p* = 0.001) and cardiac rehospitalization (HR = 1.538; 95% CI 1.069–2.214; *p* = 0.021) compared to treatment with metoprolol (Table 3).

When stratified by the LVEF, the type of BB did not affect all-cause mortality at 3 years (Table 4). However, carvedilol was associated with an increased risk of the composite arrhythmic endpoint after multivariable adjustment both in patients with an LVEF ≥ 35% (HR = 1.915; *p* = 0.012) and <35% (HR = 1.652; *p* = 0.013) (Table 4). Furthermore, cardiac rehospitalization was more frequent in patients with carvedilol and *n* LVEF < 35% (HR = 1.604; *p* = 0.039).

In line with this, the composite endpoint occurred more often in patients with carvedilol treatment, when admitted with AMI (HR = 3.642; *p* = 0.005), whereas carvedilol did not affect the risk of the composite endpoint in patients with ischemic heart disease, non-ischemic cardiomyopathy, and idiopathic VT/VF.

### 3.4. Propensity Score Matching

After propensity score matching, no significant differences regarding the distribution of ventricular tachyarrhythmias and cardiovascular risk factors were observed. Especially the rates of cardiopulmonary resuscitation and the LVEF were equally distributed among patients with carvedilol and metoprolol therapy. However, after propensity score matching, the rates of NICMP, electrophysiological examination, and VT ablation therapies were higher in patients with carvedilol therapy (Table 1; right panel). 

**Figure 3:**
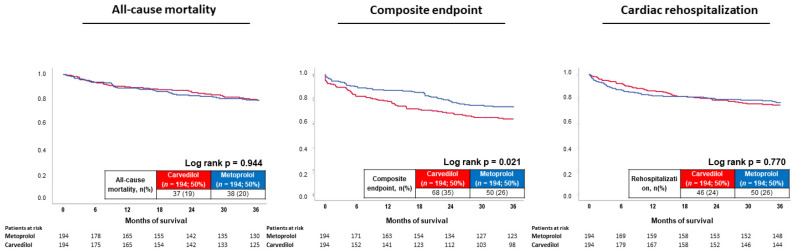
Prognostic impact of metoprolol versus carvedilol treatment on all-cause mortality (**left**), risk of the composite endpoint (i.e., recurrence of ventricular tachyarrhythmias, sudden cardiac death) (**middle**), and cardiac rehospitalization (**right**) after propensity score matching.

After propensity score matching, the type of beta-blocker had no effect on the primary endpoint all-cause mortality at 3 years (HR = 0.984; 95% CI 0.622–1.547; *p* = 0.944). In contrast, the composite arrhythmic endpoint occurred more often in patients with carvedilol therapy (HR = 1.532; 95% CI 1.063–2.207; *p* = 0.022). Finally, no prognostic impact of carvedilol and metoprolol with regard to cardiac rehospitalization was observed (HR = 1.062; 95% CI 0.711–1.585; *p* = 0.770) (Figure 3).

## 4. Discussion

The present study evaluated the prognostic impact of treatment with metoprolol compared to carvedilol on the primary endpoint of all-cause mortality, as well as on secondary endpoints, such as a composite arrhythmic endpoint (i.e., recurrence of ventricular tachyarrhythmias, appropriate ICD therapies) and cardiac rehospitalization at three years in patients surviving an index episode of ventricular tachyarrhythmias. This study suggests a comparable risk of all-cause mortality in patients treated with carvedilol vs. metoprolol. However, carvedilol was associated with an increased risk of the composite arrhythmic endpoint, which was still evident after multivariable adjustment and propensity score matching. Increased risk of the composite arrhythmic endpoint in patients with carvedilol therapy was observed both in patients with an LVEF ≥ 35% and <35%, as well as in patients admitted with AMI.

Most studies investigating the prognosis of patients treated with carvedilol as compared to metoprolol focus on patients with systolic HF, whereas heterogenous findings were reported. Thus, the prognosis of carvedilol and metoprolol was investigated within a large database including over 110,000 patients with systolic HF from 2007 to 2015. Using propensity-matched analyses, the authors found improved survival in the carvedilol group at six years [17]. In contrast, a meta-analysis with ten studies and over 30,000 patients suggested no reduction of all-cause mortality in patients with carvedilol compared to metoprolol. Accordingly, no reduction of HF hospitalization was found [18]. In line with this, the present study did not observe mortality differences between both BB therapies, whereas a significant increase of cardiac rehospitalization was observed in patients with carvedilol treatment. Within the present study, no differences regarding the rates of HF-related rehospitalization in patients treated with carvedilol and metoprolol were observed.

The anti-arrhythmic effect of carvedilol compared to metoprolol was investigated within a sub-study of the “Multicenter Automatic Defibrillator Implantation Trial With Cardiac Resynchronization Therapy” (MADIT-CRT) trial, suggesting a decrease of HF-related rehospitalization or death, as well as a significant reduction of ventricular tachyarrhythmias in patients with carvedilol therapy [19]. Furthermore, carvedilol was demonstrated to reduce the risk of inappropriate device therapies [20]. However, only patients with HF and wide QRS complexes were included in the MADIT-CRT trial. In line with this, a retrospective study by Ayan et al. included 225 ICD recipients with an LVEF ≤ 40%. During a median follow-up of 57 months, improved freedom from appropriate ICD therapies was detected in patients treated with carvedilol. In contrast, the type of BB did not affect all-cause mortality and inappropriate device therapies [21]. In those patients, a dose-dependent effect of carvedilol therapy was demonstrated [19]; however, in the present study, the recommended targeted dosage for HF treatment was not reached for carvedilol, which may explain the contrasting results in patients with ventricular tachyarrhythmias, even after adjustment for potential confounding. 

However, the present study has a different point of view, since all patients survived index episodes of ventricular tachyarrhythmias, whereas 89% of ICD recipients received ICD implantation for primary prevention of SCD in the study by Ayan et al. [21]. Contrasting results were reported within a recent study by Sessa et al. including 1424 elderly patients with HF, chronic obstructive pulmonary disease, and diabetes mellitus. Thus, carvedilol treatment was identified to increase the risk of HF-related hospitalization compared to metoprolol therapy, whereas mortality was not affected by the BB type [22]. 

Besides the type of BB, the risk of ventricular tachyarrhythmias is affected by various clinical conditions and comorbidities. For instance, in our study, the risk of the composite endpoint was decreased in patients with diabetes mellitus and AMI. Recently, we demonstrated that AMI is associated with improved prognosis in patients with ventricular tachyarrhythmias, which may be caused by improved treatment strategies, including shorter door-to-balloon times, better revascularization strategies, and an overall treatable cause for ventricular tachyarrhythmias. In line with this, the presence of diabetes mellitus and metabolic syndrome was recently shown to decrease the risk of mortality in patients with sudden cardiac arrest related to the so-called “obesity paradox”. This may be related to higher doses of cardio-protective medications (such as BBs) and higher caloric reserves in the presence of critical illness [23]. 

Besides the type of BB treatment, patients’ outcomes may also be affected by the dose of the BB therapy. Within the present study, most BB therapy was not up-titrated to the recommended daily target dose, and a major part of the patients were treated with >12.5–25% of the recommended beta-blocker target dose on index hospital discharge. However, we recently demonstrated a >12.5–25% of recommended beta-blocker target dose associated with improved long-term all-cause mortality at 3 years, whereas higher BB doses were not associated with improved outcomes [24]. Although no data are available focusing on BB doses in patients with ventricular tachyarrhythmias, the present data are in line with previous findings including patients with HF or AMI. For instance, Goldberger et al. demonstrated only 17% of AMI patients were treated with >50% of the recommended beta-blocker target dose at discharge, including 1971 patients, whereas no changes of the BB dose during the first 3 weeks following AMI were observed in 76%, suggesting up-titration is not performed according to the guideline recommendations during routine clinical care in a major part of AMI patients [25]. However, within a sub-study of the OBTAIN trial including 7057 AMI patients, a >12.5–25% of the recommended beta-blocker target dose was associated with improved risk of all-cause mortality at 1 year, whereas higher BB doses did not improve patients’ outcomes [26]. Our study confirms the findings from the OBTAIN trial with regard to patients following ventricular tachyarrhythmias. No additional benefit of higher BB doses may also be related to the dose-dependent side-effects of BB therapy, such as hypotension and bradycardia, which may further result in diminished physical activity [27].

The type of BB may also depend on patients’ symptoms, which is often not controlled for within retrospective registry data. Thus, especially carvedilol may be admitted more frequently in patients with more advanced stages of heart failure, alongside with increased New York Heart Association (NYHA) class. Interestingly, a higher NYHA class was recently shown to be an independent predictor of mortality [28]. It would therefore be of major interest to investigate the prognostic role of both BB types within further RCTs. 

Of note, the anti-arrhythmic effect of BBs may furthermore be affected by concomitant invasive treatment strategies, specifically VT ablation therapy. Thus, catheter ablation was recently shown to decrease the risk of cardiovascular death, appropriate ICD shock, hospitalization due to heart failure, or severe treatment-related complications as compared to anti-arrhythmic drugs [29]. To separate the effect of BB type and the effect of invasive therapies, further studies are necessary including patients with VT ablation therapy. 

The study has several limitations. This observational and retrospective registry-based analysis reflects a realistic picture of consecutive health-care supply to high-risk patients presenting with ventricular tachyarrhythmias. The lost to follow-up rate regarding the evaluated endpoint of all-cause mortality was minimal. Pharmacological therapies were based on discharge medication at the index event. Despite the retrospective study design, the indication for BB treatment, as well as the up-titration of BB therapy during follow-up were beyond the scope of the present study. After propensity score matching, the proportion of patients with electrophysiological examination and, specifically, VT ablation therapy was higher in the carvedilol group. Within the present study, subgroup-analyses in the VT ablation group were not possible due to the low proportion of patients undergoing VT ablation therapy. All clinical data were documented reliably by individual cardiologists during routine clinical care, being blinded to the final analyses, alleviating the use of an independent clinical event committee. Unmeasured cofounding factors (including degree HF symptoms) may not be excluded within the present study due to the retrospective study design. Cardiac rehospitalization and recurrent ventricular tachyarrhythmias were assessed at our institution only. The present results need to be re-evaluated within even larger and more representative multi-center registry data or even randomized controlled trials.

## 5. Conclusions

The present study suggests a comparable risk of all-cause mortality in patients with ventricular tachyarrhythmias treated with carvedilol and metoprolol. However, carvedilol may increase the risk of arrhythmic events. Adverse outcomes of carvedilol were still demonstrated after multivariable Cox regression analyses and propensity score matching. However, further studies, especially RCTs, will be necessary to further investigate the prognosis of different BB types for secondary prevention of ventricular tachyarrhythmias.

## Figures and Tables

**Table 1:** Baseline characteristics.

	Without Propensity Score Matching					With Propensity Score Matching				
Characteristic	Metoprolol (*n* = 879; 80%)		Carvedilol (*n* = 219; 20%)		*p*-Value	Metoprolol (*n* = 194; 50%)		Carvedilol (*n* = 194; 50%)		*p*-Value
**Age**, median (range)	65 (15–92)		68 (27–84)		**0.001**	66 (25–89)		66 (27–84)		0.208
**Male gender, *n* (%)**	645	(73)	176	(80)	**0.033**	156	(80)	158	(81)	0.796
**Ventricular tachyarrhythmias at index, *n* (%)**										
Ventricular tachycardia	542	(62)	170	(78)	**0.001**	146	(75)	150	(77)	0.633
Fast	526	(97)	161	(95)	0.236	141	(97)	147	(98)	0.450
Slow	18	(3)	9	(5)		5	(3)	3	(2)	
Monomorphic	514	(95)	162	(95)	0.811	142	(97)	145	(97)	0.766
Polymorphic	28	(5)	8	(5)		4	(3)	5	(3)	
Ventricular fibrillation	337	(38)	49	(22)	**0.001**	48	(25)	44	(23)	0.633
**Underlying cardiac disease, *n* (%)**										
Ischemic heart disease	339	(39)	112	(51)	**0.001**	108	(56)	101	(52)	0.476
STEMI	123	(14)	8	(4)	**0.001**	15	(8)	7	(4)	0.079
NSTEMI	185	(21)	28	(13)	**0.006**	19	(10)	24	(12)	0.419
Non-ischemic cardiomyopathy	37	(4)	45	(21)	**0.001**	24	(12)	43	(22)	**0.011**
Channelopathy	25	(3)	5	(2)	0.649	6	(3)	5	(3)	0.760
Idiopathic ventricular tachyarrhythmias	170	(20)	21	(10)	**0.001**	22	(12)	14	(8)	0.172
**Cardiovascular risk factors, *n* (%)**										
Arterial hypertension	536	(61)	137	(63)	0.668	133	(69)	122	(63)	0.239
Diabetes mellitus	210	(24)	82	(37)	**0.001**	66	(34)	71	(37)	0.595
Hyperlipidemia	273	(31)	86	(39)	**0.020**	67	(35)	79	(41)	0.209
Smoking	291	(33)	68	(31)	0.562	60	(31)	60	(31)	1.000
Cardiac family history	106	(12)	27	(12)	0.913	29	(15)	25	(13)	0.557
**Prior medical history, *n* (%)**										
Beta-blocker	216	(25)	70	(32)	**0.026**	66	(34)	63	(33)	0.746
ACE inhibitor	185	(21)	53	(24)	0.311	67	(35)	47	(24)	0.026
ARB	44	(45)	21	(10)	**0.010**	8	(4)	21	(11)	0.012
Statin	168	(19)	48	(22)	0.350	47	(24)	43	(22)	0.630
Amiodarone	19	(2)	9	(4)	0.102	10	(5)	9	(5)	0.814
Digitalis	50	(6)	28	(13)	**0.001**	23	(12)	47	(14)	0.544
Aldosterone antagonist	30	(3)	12	(6)	0.154	13	(7)	12	(6)	0.836
**Comorbidities at index stay, *n* (%)**										
Prior myocardial infarction	226	(26)	75	(34)	**0.011**	66	(34)	71	(37)	0.595
Prior coronary artery disease	362	(41)	115	(53)	**0.002**	110	(57)	107	(55)	0.759
Prior heart failure	193	(22)	108	(49)	**0.001**	98	(51)	99	(51)	0.919
Atrial fibrillation	249	(28)	81	(37)	**0.012**	68	(35)	69	(36)	0.915
Idiopathic ventricular tachyarrhythmias										
Cardiopulmonary resuscitation	326	(37)	43	(19)	**0.001**	45	(23)	37	(19)	0.598
In hospital	109	(12)	16	(7)		18	(9)	14	(7)	
Out of hospital	217	(25)	27	(12)		27	(14)	23	(12)	
Chronic kidney disease						96	(50)	95	(49)	0.919
**Coronary angiography, *n* (%)**	655	(75)	137	(63)	**0.001**	127	(66)	125	(64)	0.831
No evidence of CAD	147	(22)	48	(35)	**0.003**	33	(26)	46	(37)	**0.031**
1-vessel disease	174	(27)	21	(15)		30	(24)	17	(14)	
2-vessel disease	147	(22)	34	(25)		23	(18)	32	(26)	
3-vessel disease	187	(29)	34	(25)		41	(32)	30	(24)	
Chronic total occlusion	120	(18)	34	(25)	0.081	31	(24)	30	(24)	0.940
Presence of CABG	88	(13)	23	(17)	0.304	24	(19)	20	(16)	0.545
PCI	318	(49)	32	(23)	**0.001**	41	(32)	28	(22)	0.079
**LVEF, *n* (%)**										
>55%	248	(33)	12	(6)	**0.001**	21	(11)	12	(6)	0.291
54–45%	137	(18)	18	(9)		13	(7)	18	(9)	
44–35%	143	(19)	44	(22)		36	(19)	42	(22)	
<35%	216	(29)	126	(63)		124	(64)	122	(63)	
No evidence of LVEF	354	-	19	-						
**Cardiac therapies at index, *n* (%)**										
Electrophysiological examination	240	(27)	95	(43)	**0.001**	62	(32)	90	(46)	**0.004**
VT ablation therapy	44	(5)	24	(11)	**0.001**	9	(5)	23	(12)	**0.010**
**Presence of an ICD at discharge, *n* (%)**	399	(45)	177	(81)	**0.001**	161	(83)	161	(83)	1.000
**Medication at discharge, *n* (%)**										
ACE inhibitor	613	(70)	159	(73)	0.419	151	(78)	137	(71)	0.104
ARB	81	(9)	34	(16)	**0.007**	16	(9)	33	(17)	**0.012**
Statin	621	(71)	137	(63)	**0.019**	130	(67)	122	(63)	0.395
Amiodarone	119	(14)	57	(26)	**0.001**	48	(25)	48	(25)	1.000
Digitalis	87	(10)	60	(27)	**0.001**	33	(17)	52	(27)	0.020
Aldosterone antagonist	81	(9)	51	(23)	**0.001**	38	(20)	46	(24)	0.324

ACE, angiotensin converting enzyme; ARB, angiotensin receptor blocker; CABG, coronary artery bypass grafting; CAD, coronary artery disease; COPD, chronic obstructive pulmonary disease; LVEF, left ventricular ejection fraction; (N)STEMI, (non-)ST segment myocardial infarction; PCI, percutaneous coronary intervention; SEM, standard error of mean; VT, ventricular tachycardia. Bold type indicates *p* < 0.05.

**Table 2:** Primary and secondary endpoints, follow-up data.

	Without Propensity Score Matching					With Propensity Score Matching				
Characteristics	Metoprolol (*n* = 879; 80%)		Carvedilol (*n* = 219; 20%)		*p*-Value	Metoprolol (*n* = 194; 50%)		Carvedilol (*n* = 194; 50%)		*p*-Value
**Primary endpoint, *n* (%)**										
All cause-mortality, at 3 years	144	(16)	43	(20)	0.235	38	(20)	37	(19)	0.944
**Secondary endpoints, *n* (%)**										
Cardiac rehospitalization, at 3 years	120	(14)	54	(25)	**0.001**	46	(24)	50	(26)	0.077
Ventricular tachycardia	32	(4)	6	(3)	**0.006**	6	(3)	6	(3)	0.314
Ventricular fibrillation	12	(1)	2	(0.9)		2	(1)	2	(1)	
Acute myocardial infarction	4	(0.5)	0	(0)		2	(1)	0	(0)	
Acute heart failure	34	(4)	22	(10)		12	(6)	20	(10)	
Inappropriate device therapy	22	(3)	14	(6)		6	(3)	12	(6)	
Other	16	(2)	10	(5)		12	(6)	10	(5)	
Composite endpoint (recurrent ventricular tachyarrhythmias, appropriate ICD therapy), at 3 years	149	(17)	71	(32)	**0.001**	50	(26)	68	(35)	**0.022**
Recurrent ventricular tachyarrhythmias without ICD therapy	29	(19)	6	(8)	**0.034**	8	(16)	6	(9)	0.234
Appropriate ICD therapy	118	(81)	65	(92)		42	(84)	62	(91)	
**Follow-up times, *n* (%)**										
Hospitalization time; days (median (IQR))	14 (8–23)		12 (9–25)		**0.007**	15 (8–23)		12 (9–25)		0.450
ICU time; days (median (IQR))	3 (0–8)		2 (0–7)		0.382	3 (0–7)		2 (0–7)		0.440
Survival time; days (mean; median (range))	1908; 1724 (3–5106)		1992; 1792 (18–5091)		**0.011**	1909; 1790 (15–5106)		2040; 1784 (20–5091)		0.364

ICU, invasive care unit; IQR, interquartile range. Level of significance *p* ≤ 0.05. Bold type indicates *p* ≤ 0.05.

**Table 3:** Multivariable Cox regression analyses within the entire study cohort.

Endpoint	All-Cause Mortality			Composite Endpoint			Cardiac Rehospitalization		
	HR	95% CI	*p*-Value	HR	95% CI	*p*-Value	HR	95% CI	*p*-Value
Age (decades)	1.522	1.281–1.808	0.001	1.113	0.979–1.266	0.102	1.011	0.876–1.166	0.882
Males	1.010	0.685–1.488	0.961	1.249	0.868–1.798	0.230	1.451	0.931–2.261	0.100
Diabetes	1.992	1.443–2.750	0.001	0.666	0.473–0.938	0.020	0.903	0.628–1.298	0.582
Prior heart failure	1.349	0.956–1.903	0.088	1.205	0.883–1.646	0.240	1.555	1.088–2.221	0.015
Chronic kidney disease	1.721	1.238–2.393	0.001	1.079	0.807–1.443	0.607	1.040	0.748–1.446	0.817
AMI	0.805	0.530–1.223	0.310	0.543	0.354–0.832	0.005	1.012	0.672–1.524	0.954
AF	1.267	0.913–1.759	0.156	1.169	0.864–1.581	0.311	1.466	1.047–2.054	0.026
LVEF < 35%	1.383	0.972–1.967	0.071	1.514	1.102–2.081	0.011	1.630	1.135–2.342	0.008
Coronary artery disease	1.029	0.683–1.552	0.890	0.815	0.587–1.132	0.223	1.373	0.897–2.102	0.145
Carvedilol vs. metoprolol	0.811	0.550–1.194	0.288	1.726	1.261–2.364	0.001	1.538	1.069–2.214	0.021

AF; atrial fibrillation; AMI; acute myocardial infarction; CI; confidence interval; HR; hazards ratio; LVEF, left ventricular ejection faction. Level of significance *p* ≤ 0.05. Bold type indicates statistical significance.

**Table 4:** Multivariable Cox regression analyses within important subgroups.

Endpoint	All-Cause Mortality			Composite Endpoint			Cardiac Rehospitalization		
	HR	95% CI	*p*-Value	HR	95% CI	*p*-Value	HR	95% CI	*p*-Value
LVEF ≥ 35%	0.537	0.239–1.206	0.132	1.915	1.152–3.184	0.012	1.400	0.726–2.698	0.315
LVEF < 35%	1.002	0.632–1.587	0.994	1.652	1.110–2.459	0.013	1.604	1.024–2.513	0.039
Ischemic heart disease	1.038	0.647–1.665	0.878	1.437	0.940–2.197	0.095	1.692	1.062–2.695	0.027
Acute myocardial infarction	0.412	0.114–1.481	0.174	3.642	1.478–8.975	0.005	1.588	1.120–5.983	0.026
Non-ischemic cardiomyopathy	0.496	0.179–1.371	0.176	1.361	0.651–2.847	0.412	0.589	0.237–1.463	0.254
Idiopathic ventricular tachyarrhythmias	0.110	0.009–1.295	0.079	2.631	0.916–7.558	0.072	0.889	0.095–8.307	0.918

HR; hazards ratio; LVEF, left ventricular ejection faction. Multivariable Cox regression models were adjusted for age, gender, diabetes mellitus, prior heart failure, chronic kidney disease, acute myocardial infarction, atrial fibrillation, LVEF < 35%, coronary artery disease, and carvedilol vs. metoprolol therapy. Level of significance *p* ≤ 0.05. Bold type indicates statistical significance.

## Data Availability

Not applicable.
